# Association of common polymorphisms in known susceptibility genes with rheumatoid arthritis in a Slovak population using osteoarthritis patients as controls

**DOI:** 10.1186/ar2699

**Published:** 2009-05-15

**Authors:** Klaus Stark, Jozef Rovenský, Stanislava Blažičková, Hans Grosse-Wilde, Stanislav Ferencik, Christian Hengstenberg, Rainer H Straub

**Affiliations:** 1 Department of Internal Medicine II, University Hospital Regensburg, Franz-Josef-Strauss-Allee 11, 93042 Regensburg, Germany; 2 National Institute of Rheumatic Diseases, Nabr. I. Krasku 4, 921 23 Piešt'any, Slovakia; 3 Institute of Immunology, University Hospital of Essen, Virchowstrasse 179, 45122 Essen, Germany; 4 Department of Internal Medicine I, University Hospital Regensburg, Franz-Josef-Strauss-Allee 11, 93042 Regensburg, Germany

## Abstract

**Introduction:**

Both genetic and environmental factors contribute to rheumatoid arthritis (RA), a common and complex autoimmune disease. As well as the major susceptibility gene *HLA-DRB1*, recent genome-wide and candidate-gene studies reported additional evidence for association of single nucleotide polymorphism (SNP) markers in the *PTPN22*, *STAT4*, *OLIG3/TNFAIP3 *and *TRAF1/C5 *loci with RA. This study was initiated to investigate the association between defined genetic markers and RA in a Slovak population. In contrast to recent studies, we included intensively-characterized osteoarthritis (OA) patients as controls.

**Methods:**

We used material of 520 RA and 303 OA samples in a case-control setting. Six SNPs were genotyped using TaqMan assays. *HLA-DRB1 *alleles were determined by employing site-specific polymerase chain reaction (PCR) amplification.

**Results:**

No statistically significant association of *TRAF1/C5 *SNPs rs3761847 and rs10818488 with RA was detected. However, we were able to replicate the association signals between RA and *HLA-DRB1 *alleles, *STAT4 *(rs7574865), *PTPN22 *(rs2476601) and *OLIG3/TNFAIP3 *(rs10499194 and rs6920220). The strongest signal was detected for HLA-DRB1*04 with an allelic *P *= 1.2*10^-13 ^(OR = 2.92, 95% confidence interval (CI) = 2.18 – 3.91). Additionally, SNPs rs7574865_*STAT4 *_(*P *= 9.2*10^-6^; OR = 1.71, 95% CI = 1.35 – 2.18) and rs2476601_*PTPN22 *_(*P *= 9.5*10^-4^; OR = 1.67, 95% CI = 1.23 – 2.26) were associated with susceptibility to RA, whereas after permutation testing *OLIG3/TNFAIP3 *SNPs rs10499194 and rs6920220 missed our criteria for significance (*P*_corr _= 0.114 and *P*_corr _= 0.180, respectively).

**Conclusions:**

In our Slovak population, *HLA-DRB1 *alleles as well as SNPs in *STAT4 *and *PTPN22 *genes showed a strong association with RA.

## Introduction

Susceptibility to rheumatoid arthritis (RA) is influenced by both environmental and genetic determinants with a concordance rate in monozygotic twins between 12% and 30% and a λ_*s *_ranging from three to seven [1]. One of the first known genetic loci responsible for susceptibility to RA was found within the major histocompatibility complex, namely immune response genes in the human leukocyte antigen (HLA) class II region [2].

Recent genome-wide association studies have confirmed known and identified new genetic determinants of RA [3]. The well studied associations with *HLA-DRB1 *and *PTPN22 *explain about 50% of the genetic contribution to RA disease susceptibility [4]. For other polymorphisms, strong associations with RA were demonstrated, namely for a single nucleotide polymorphism (SNP) in the *STAT4 *gene, for two independent alleles at chromosome 6q23 near *OLIG3 *and *TNFAIP3 *genes, and for SNPs near *TRAF1 *and *C5 *genes [5-9].

In contrast to recent studies, we performed a replication study of seven genetic polymorphisms in Slovak patients with chronic RA as cases and with chronic osteoarthritis (OA) as controls. RA and OA share some features of pathology, but in detail seem to be quite different entities [10-13]. For a functional variant in the *GDF5 *gene, it was recently shown that risk of both RA and OA is increased [14,15]. Therefore, more genetic markers might be involved in both diseases.

To the best of our knowledge, this is the first study aimed at examining a genetic association in a RA-OA case-control setting in a Slovak population.

## Materials and methods

### Study participants

A total of 520 Slovak individuals (87 males, 433 females) with the diagnosis of RA were recruited to this study. All RA cases fulfilled the diagnostic features based on the established American College of Rheumatology criteria [13]. Controls (60 males, 243 females) were unrelated individuals from Slovakia who did not have any indication of RA but were affected by OA and intensively characterized. Further phenotypic details are shown in Table 1. Our study population did not differ in gender between RA cases and RA-free OA controls. Controls with OA are significantly older but free of RA symptoms and are rheumatoid factor (RF) negative. Both serum anti-cyclic citrullinated peptide (CCP) and C-reactive protein levels are significantly lower in OA than in RA cases (Table 1).

**Table 1:** Characteristics of study sample

Variable	RA cases (n = 520)	RA-free OA controls (n = 303)	*P*
Gender, % female (n)	83.3 (433)	80.2 (243)	ns
Age at inclusion, years (range)	51.6 ± 11.2 (19 to 80)	57.9 ± 13.5 (21 to 83)	< 0.0001
Age of onset, years	40.8 ± 12.7	50.7 ± 12.8	< 0.0001
Duration of disease, years	10.8 ± 8.3	7.2 ± 6.8	< 0.0001
RF, IU/ml	149.8 ± 67.2	-	-
RF-positive, % (n)	53.8 (280)	-	-
anti-CCP antibody, RU/ml^a^	67.5 ± 53.7	1.5 ± 6.4	< 0.0001
anti-CCP positive, % (n)^b^	78.6 (239)	3.2 (4)	< 0.0001
CRP, μg/ml	19.6 ± 23.7	5.1 ± 9.2	< 0.0001

Values denote means ± standard deviations unless indicated otherwise.

CCP = cyclic citrullinated peptide; CRP = C-reactive protein; ns = not significant; RF = rheumatoid factor.

^a ^anti-CCP antibody serum level was determined in 428 individuals.

^b ^Values below 4.2 RU/ml were considered as anti-CCP negative.

Measurement of antibody against CCP was carried out using an anti-CCP-ELISA (Euroimmun, Lübeck, Germany) following the manufacturer's instructions. From a total of 428 individuals (304 RA patients, 124 OA patients) anti-CCP antibodies were determined. Values less than 4.2 RU/ml were considered as anti-CCP negative. No value exceeded the proposed linear range of up to 196 RU/ml. The RF was determined by standard techniques in the Laboratories of the National Institute of Rheumatic Diseases, Piestany, Slovakia.

Written consent was obtained from the patients according to the current Declaration of Helsinki. The study was approved by the Ethical Committee of the National Institute of Rheumatic Diseases, Piestany, Slovakia.

### Marker selection and genetic analyses

SNPs in or near the genes *PTPN22*, *STAT4*, *OLIG3*/*TNFAIP3*, and *TRAF1*/*C5 *were selected from recent genome-wide association studies with replication studies and candidate-gene approaches (Table 2) [4-9].

**Table 2:** SNP markers used in analysis

SNP	Position^a^	Major allele (1)	Minor allele (2)	Gene/function
rs2476601	chr 1: 114,179,091	G	A	*PTPN22*/R620W
rs7574865	chr 2: 191,672,878	G	T	*STAT4*/Intron
rs10499194	chr 6: 138,044,330	C	T	intergenic between *OLIG3 *and *TNFAIP3*
rs6920220	chr 6: 138,048,197	G	A	intergenic between *OLIG3 *and *TNFAIP3*
rs3761847	chr 9: 122,730,060	A	G	intergenic between *TRAF1 *and *C5*
rs10818488	chr 9: 122,744,908	G	A	intergenic between *TRAF1 *and *C5*

^a ^according to NCBI build 36.3.

SNP = single nucleotide polymorphism.

Genomic DNA was isolated from whole blood samples using the PureGene DNA Blood Kit (QIAGEN, Hilden, Germany). DNA samples were genotyped using 5' exonuclease TaqMan^® ^technology (Applied Biosystems, Foster City, CA, USA), as recently described [16]. In brief, for each genotyping experiment 10 ng DNA was used in a total volume of 5 μl containing 1 × TaqMan^® ^Genotyping Master Mix (Applied Biosystems Foster City, CA, USA). PCR and post-PCR endpoint plate read was carried out according to the manufacturer's instructions using the Applied Biosystems 7900 HT Real-Time PCR System (Foster City, CA, USA). Sequence Detection System software version 2.3 (Applied Biosystems, Foster City, CA, USA) was used to assign genotypes applying the allelic discrimination test. Case and control DNA was genotyped together on the same plates with duplicates of samples (15%) to assess intraplate and interplate genotype quality. No genotyping discrepancies were detected. Assignment of genotypes was performed by a person with no knowledge of the proband's affection status.

*HLA-DRB1 *genotyping was carried out using PCR with *HLA-DRB1 *low-resolution exon 2 sequence-specific primers as previously described [17]. Absence or presence of *HLA-DRB1 *specific products was visualized by agarose gel electrophoresis, photographed, and documented.

*HLA-DRB1 *alleles were classified according to the nomenclature proposed by the World Health Organization Nomenclature Committee for factors of the HLA system [18]. For shared epitope (SE) association with RA, the classification system from de Vries was employed [19]. Due to frequencies below 1% for protective *HLA-DRB1 *allele *0402 and neutral alleles *0403, *0406, and *0407, we did not analyse the *04 group in high resolution and considered *04 in total as SE [20]. With only three alleles in our study population (one in OA controls and two in RA cases), HLA-DRB1*0103 was not used as a separate genotype and therefore *01 was also considered as SE in total.

### Statistical analyses

To determine whether the genotypes of cases and controls of all SNPs deviated from Hardy-Weinberg equilibrium, actual and predicted genotype counts of both groups were compared by an exact test [21]. Differences between dichotomous traits were calculated employing a chi-squared test. Genotypes were coded for both dominant and recessive effects (genotype 22 + 12 versus 11 and genotype 22 versus 11 + 12, respectively, with the minor allele coded as 2). The additive genetic model was calculated using Armitage's trend test [22]. To test for epistatic interaction between SNP markers a logistic regression model based on allele dosage for each SNP was carried out. Differences in continuous variables between groups were calculated using a two-tailed *t*-test for normally distributed values or using the non-parametric Wilcoxon rank-sum test for variables failing normal distribution as determined by the Shapiro-Wilk test. Multiple logistic regression analysis was used to examine the association between SNPs and RA with *HLA-DRB1 *genotypes as covariates. Prevalence odds ratios (OR) with their 95% confidence intervals (CI) were reported. Correction for multiple testing was carried out using the Bonferroni adjustment. For *post-hoc *power calculation Fisher's exact test was used. A one-sided *P *≤ 0.05 was considered statistically significant.

Association analyses were performed using JMP 7.0.2 (SAS Institute Inc, Cary, NC, USA) and PLINK v1.04 [23,24]. For analysis of linkage disequilibrium (LD) and for permutation testing HaploView v4.1 was employed [25,26]. Power analysis was carried out using G*Power 3.0.10 [27,28].

## Results

### Genetic analyses – SNP marker association

We analyzed six SNPs with prior evidence of association with RA in genome-wide association studies and candidate-gene approaches, namely in or near the genes *PTPN22*, *STAT4*, *OLIG3*/*TNFAIP3*, and *TRAF1*/*C5 *(Tables 2 and 3) [4-9]. Additionally, *HLA-DRB1 *alleles were determined in low resolution and classified in respect to the SE [see Table S1 in Additional data file 1].

**Table 3:** Power analysis of SNP markers

SNP	Published OR^a^	Published MAF in controls	Ref	Current study's MAF in controls	Power^b^
rs2476601	1.98	0.10	[4]	0.108	94.5%
rs7574865	1.27	0.22	[5]	0.202	35.9%
rs10499194	0.75	0.21 to 0.31	[6]	0.315	53.1%
rs6920220	1.22	0.21 to 0.22	[7]	0.154	23.4%
rs3761847	1.32	0.37 to 0.45	[8]	0.387	57.0%
rs10818488	1.26	0.44	[9]	0.390	44.4%

OR = odds ratio; MAF = minor allele frequency; Ref = reference; SNP = single nucleotide polymorphism.

^a ^combination of initial finding and replication (when available) in the cited study; effects from minor allele.

^b ^Power was calculated for published OR and MAF in controls from the present study (Table 4) with 520 cases and 303 controls assuming a one-tailed *P *= 0.05.

For all six SNP markers analyzed, call rates were greater than 98.5% and no deviation from the Hardy-Weinberg equilibrium was observed both in RA cases and RA-free OA controls (Table 4). Between *TRAF1 *and *C5 *SNPs on chromosome 9 (rs3761847 and rs10818488, respectively) strong LD exist with an *r^2 ^*value of 0.99. Weak LD (*r*^2^= 0.08) was detected between the two SNPs on chromosome 6 (rs10499194 and rs6920220), whereas the other SNPs are unlinked (*r*^2 ^= 0) and lie on different chromosomes.

**Table 4:** SNP characteristics in RA-OA case-control sample

	RA case genotypes					RA-free OA control genotypes				
		
SNP	11	12	22	MAF	*P *(HWE)	11	12	22	MAF	*P *(HWE)
rs2476601	356	144	14	0.167	1	239	61	2	0.108	0.551
rs7574865	259	205	54	0.302	0.175	196	87	17	0.202	0.104
rs10499194	281	200	37	0.264	0.910	149	116	37	0.315	0.062
rs6920220	324	175	16	0.201	0.218	213	78	7	0.154	1
rs3761847	186	243	87	0.404	0.648	117	133	49	0.387	0.275
rs10818488	186	240	85	0.401	0.645	116	134	50	0.390	0.278

HWE = Hardy-Weinberg equilibrium; MAF = minor allele frequency; OA = osteoarthritis; RA = rheumatoid arthritis; SNP = single nucleotide polymorphism. Numbers of genotypes (11, 12, 22) according to alleles from Table 2.

A strong association between two SNPs (rs7574865_*STAT4 *_and rs2476601_*PTPN22*_) and RA was detected, whereas for *OLIG3*/*TNFAIP3 *SNPs rs10499194 and rs6920220 nominal association was found. *TRAF1/C5 *SNPs rs3761847 and rs10818488 did not reach statistical significance in our study population (Table 5). However, OR for all SNPs are shifted in the same direction as previously published (Table 3). After correction for multiple testing (six SNPs), allelic *P*-values were still significant for rs7574865_*STAT4 *_and rs2476601_*PTPN22 *_(*P*_corr _= 5.5 × 10^-5 ^and *P*_corr _= 5.7 × 10^-3^, respectively), but not for the other four SNPs (Table 5). Different genetic models revealed no considerable stronger association than observed by comparison of allele frequencies [see Table S2 in Additional data file 1]. After 100,000 permutation testings, rs7574865_*STAT4 *_still showed the strongest association signal (*P *= 8.0 × 10^-5^) with rs2476601_*PTPN22 *_(*P *= 5.9 × 10^-3^). The other SNPs failed to reach a level of statistical significance (rs6920220_*OLIG3/TNFAIP3*_, *P *= 0.105; rs10499194_*OLIG3/TNFAIP3*_, *P *= 0.152; rs3761847_*TRAF1/C5*_, *P *= 0.966; rs10818488_*TRAF1/C5*_, *P *= 0.996).

**Table 5:** SNP association analysis results in RA-OA case-control sample

	Allelic	Allelic	Allelic OR	
SNP	*P*	*P *corr.^a^	(95% CI)	Locus
rs2476601	9.5 × 10^-4^	5.7 × 10^-3^	1.67 (1.23 to 2.26)	*PTPN22*
rs7574865	9.2 × 10^-6^	5.5 × 10^-5^	1.71 (1.35 to 2.18)	*STAT4*
rs10499194	0.030	0.180	0.78 (0.63 to 0.98)	*OLIG3/TNFAIP3*
rs6920220	0.019	0.114	1.38 (1.05 to 1.80)	*OLIG3/TNFAIP3*
rs3761847	0.480	1	1.08 (0.88 to 1.32)	*TRAF1/C5*
rs10818488	0.657	1	1.05 (0.85 to 1.29)	*TRAF1/C5*

^a ^Bonferroni correction for six SNPs tested.

CI = confidence interval; OA = osteoarthritis; OR = odds ratio; RA = rheumatoid arthritis; SNP = single nucleotide polymorphism.

Analysis of epistasis revealed no significant interaction between the six SNPs (best *P *= 0.063 for epistatic interaction between rs7574865_*STAT4 *_and rs2476601_*PTPN22*_, and between rs7574865_*STAT4 *_and rs10499194_*OLIG3/TNFAIP3 *_with *P *= 0.073). In particular, the two SNPs localized on chromosome 6 between *OLIG3 *and *TNFAIP3 *genes (rs10499194 and rs6920220) showed no interaction (*P *= 0.425).

Gender-specific analyses showed no association between the six SNPs and RA in the male subgroup (87 cases, 60 controls) [see Table S3 in Additional data file 1]. However, in the female subgroup (433 cases, 243 controls) the SNPs rs7574865_*STAT4*_, rs2476601_*PTPN22*_, and rs10499194_*OLIG3/TNFAIP3 *_were associated with susceptibility to RA [see Table S4 in Additional data file 1], even after correction for multiple testing (*P*_corr _= 2.8 × 10^-5^, *P*_corr _= 9.0 × 10^-3 ^and *P*_corr _= 0.037, respectively).

In a subset analysis of RA samples stratified to RF status, no association between SNPs and RF status were found by comparison of RF-positive and RF-negative RA cases [see Table S5 in Additional data file 1]. In contrast, RF-positive and RF-negative RA cases compared with OA controls showed effects for SNPs rs7574865_*STAT4 *_and rs2476601_*PTPN22 *_in the same order of magnitude (OR = 1.62 to 1.74) as the whole RA sample [see Tables S6 and S7 in Additional data file 1].

To test for an influence of serum anti-CCP antibody on RA susceptibility, association analyses between SNPs and RA were carried out in stratified subgroups [see Tables S8 to S10 in Additional data file 1]. Only *PTPN22 *SNP rs2476601 reached statistical significance after correction for multiple testing when comparing anti-CCP-positive RA patients with OA controls (*P*_corr _= 2.5 × 10^-3^).

### Genetic analyses – HLA allele association

*HLA-DRB1 *alleles were determined in 795 individuals (96.6%). Borderline deviation from Hardy-Weinberg equilibrium was found for HLA-DRB1*01 in controls and for *07 in cases (Table 6).

**Table 6:** *HLA-DRB1 *allele distribution in RA-OA case-control sample

	RA case genotypes^b^					RA-free OA control genotypes^b^				
		
*HLA-DRB1 *allele^a^	0	1	2	MAF	*P *(HWE)	0	1	2	MAF	*P *(HWE)
*01	350	139	7	0.154	0.121	223	75	1	0.129	0.040
*03	412	81	3	0.088	1	251	45	3	0.085	0.459
*04	268	192	36	0.266	0.819	234	64	1	0.110	0.229
*07	414	82	0	0.083	0.038	224	69	6	0.136	0.804
*08	471	25	0	0.025	1	279	20	0	0.033	1
*09	485	11	0	0.011	1	296	3	0	0.005	1
*10	466	30	0	0.030	1	294	5	0	0.008	1
*11	403	88	5	0.099	0.804	229	68	2	0.120	0.279
*12	486	10	0	0.010	1	282	17	0	0.028	1
*13	434	59	3	0.066	0.457	226	69	4	0.129	0.799
*14	475	21	0	0.021	1	283	15	1	0.028	0.209
*15	417	74	5	0.085	0.381	228	68	3	0.124	0.593
*16	435	60	1	0.063	0.711	262	36	1	0.064	1

^a ^Allele numbering according to Table S1 in Additional data file 1.

^b ^Numbers indicate counts of rare alleles.

HWE = Hardy-Weinberg equilibrium; MAF = minor allele frequency; OA = osteoarthritis; RA = rheumatoid arthritis.

Except for HLA-DRB1*01, all association results confirmed our assumption of *HLA-DRB1 *allele classification [see Table S1 in Additional data file 1] (Table 7). Highest signals for risk association to RA were observed for HLA-DRB1*04 and *10 (Table 7). HLA-DRB1*07, *12, *13, and *15 showed protective effects (Table 7). After correction for multiple testing (13 tests), alleles *04, *07, and *13 still remained significant (*P*_corr _= 2.0 × 10^-12^, *P*_corr _= 0.010 and *P*_corr _= 2.3 × 10^-4^, respectively).

**Table 7:** *HLA-DRB1 *allele association analysis results in RA-OA case-control sample

	Allelic	Allelic OR	
*HLA-DRB1 *allele^a^	*P*	(95% CI)	Classification^b^
*01	0.162	1.25 (0.92 to 1.66)	N
*03	0.868	1.03 (0.72 to 1.48)	N
*04	1.2 × 10^-13^	2.92 (2.18 to 3.91)	SE
*07	7.7 × 10^-4^	0.58 (0.41 to 0.80)	P
*08	0.337	0.75 (0.41 to 1.36)	N
*09	0.209	2.22 (0.62 to 8.00)	N
*10	4.0 × 10^-3^	3.70 (1.43 to 9.59)	SE
*11	0.177	0.81 (0.58 to 1.11)	N
*12	6.1 × 10^-3^	0.35 (0.16 to 0.77)	P
*13	1.8 × 10^-5^	0.47 (0.34 to 0.67)	P
*14	0.359	0.74 (0.39 to 1.41)	N
*15	0.012	0.66 (0.47 to 0.92)	P
*16	0.934	0.98 (0.65 to 1.49)	N

^a ^Allele numbering according to Table S1 in Additional data file 1.

^b ^See Table S1 in Additional data file 1.

CI = confidence interval; N = neutral allele; OA = osteoarthritis; OR = odds ratio; P = protective allele; RA = rheumatoid arthritis; SE = shared epitope allele.

In gender-specific analyses, we found associations to RA susceptibility in our male subgroup for HLA-DRB1*04 and protective effects for alleles *12 and *13 [see Table S11 in Additional data file 1]. However, after correction for multiple testing, only allele *13 achieved marginal statistical significance (*P*_corr _= 0.043). The female subgroup showed almost the same pattern of association as the whole population, except for alleles *11 and *12 [see Table S12 in Additional data file 1], whereas after correction for multiple testing, alleles *04, *07, and *13 still met our criteria for significance (*P*_corr _= 6.9 × 10^-11^, *P*_corr _= 2.1 × 10^-3^, and *P*_corr _= 0.014, respectively). In both genders, no inflation of association signals was caused by deviation from Hardy-Weinberg equilibrium [see Tables S11 and S12 in Additional data file 1].

Additionally, we carried out a subset analysis of RA samples stratified to RF status. Association between RA and *HLA-DRB1 *alleles *04, *07, and *11 was detected by comparison of RF-positive and RF-negative RA cases [see Table S13 in Additional data file 1], whereas after correction for multiple testing, alleles *04 and *07 still met our criteria for significance (*P*_corr _= 0.018 for both alleles). Comparison of RF-positive cases with OA controls showed association signals for *HLA-DRB1 *alleles *04, *07, *10, *11, *12, and *13, after correction for multiple testing alleles *11 and *12 failed significance [see Table S14 in Additional data file 1]. Alleles *04, *13, and *15 were associated with RA when comparing RF-negative cases with OA controls [see Table S15 in Additional data file 1], but only risk allele *04 met significance criteria after correction for multiple testing (*P*_corr _= 5.3 × 10^-5^).

Stratification for serum anti-CCP antibody showed risk effect of HLA-DRB1*04 and protective effect of allele *13 in RA patients [see Table S16 in Additional data file 1] even after correction for multiple testing (*P*_corr _= 0.025 and *P*_corr _= 0.036, respectively). Comparison of anti-CCP-positive RA cases with anti-CCP-negative OA controls revealed several association signals, whereas anti-CCP-negative RA cases did not [see Tables S17 and S18 in Additional data file 1].

Assuming a dominant genetic model for *HLA-DRB1 *alleles, we carried out a multiple logistic regression analysis to test for interactions between *HLA-DRB1 *alleles and the six SNPs. Taking into account all 13 *HLA-DRB1 *alleles, a significant association between RA and rs7574865_*STAT4 *_as well as rs2476601_*PTPN22 *_remained (*P *= 2.8 × 10^-4 ^and *P *= 1.9 × 10^-3^, respectively), whereas the other SNPs failed to reach the level of statistical significance (rs10499194_*OLIG3/TNFAIP3*_, *P *= 0.140; rs6920220_*OLIG3/TNFAIP3*_, *P *= 0.079; rs3761847_*TRAF1/C5*_, *P *= 0.771; rs10818488_*TRAF1/C5*_, *P *= 0.897). After adjustment for only risk *HLA-DRB1 *alleles *04 and *10, for four SNPs signficant association was detected (rs7574865_*STAT4*_, *P *= 1.4 × 10^-5^; rs2476601_*PTPN22*_, *P *= 1.2 × 10^-3^; rs6920220_*OLIG3/TNFAIP3*_, *P *= 4.6 × 10^-3^; rs10499194_*OLIG3/TNFAIP3*_, *P *= 0.017) but not for rs3761847_*TRAF1/C5 *_and rs10818488_*TRAF1/C5 *_(*P *= 0.790 and *P *= 0.943, respectively).

## Discussion

This study investigated the relation between known susceptibility alleles and RA in a Slovak population. In contrast to recent studies, we compared RA cases with gender-matched OA controls. Therefore, this paper is the first to analyze the differences between RA and OA for known high-risk genetic polymorphisms.

Since the 1970s it has been known that variants in the *HLA *region on chromosome 6p21.3 are associated with RA [29]. In our study, the main effect to RA risk came from HLA-DRB1*04 allele. Additionally, we found protective effects of HLA-DRB1*07 and *13 in the whole study group. However, common SNP markers in genes *PTPN22 *and *STAT4 *also contributed to RA susceptibility, but no other SNPs analyzed. It is noteworthy, that, in contrast to other studies, *STAT4 *SNP rs7574865 showed higher significance than *PTPN22 *SNP rs2476601. One explanation may be our study design. By comparing RA with OA patients, genes with opposing effects will show higher OR.

For SNPs rs3761847 and rs10818488, localized between *TRAF1 *and *C5 *genes, we were not able to find a statistically significant association with RA. Recently, re-evaluation of RA susceptibility genes in the Wellcome Trust Case Control Consortium study revealed very moderate effect sizes for SNPs in the *TRAF1/C5 *genomic region (OR = 1.08) [30]. The effect of *TRAF1/C5 *alleles may have been over-estimated in the initial study ('winner's curse'). Therefore, in replication studies, the moderate effects have to be the basis for analysis.

The power to detect association in our study was only 12% (minor allele frequency = 39%, assumed OR = 1.08, one-tailed *P *= 0.05). Hence, both missing power and ethnicity could explain the non-replication of these associations with RA in our Slovak population. For example, minor allele frequency for rs10818488 in controls is lower in our study (0.39) compared with published data in sample sets from the Netherlands, Sweden, and the USA (0.44) [9]. Another reason could be the pathophysiological identity in genetic susceptibility between RA and OA. Our study is designed to work out specific genetic differences to RA susceptibility in comparison to OA. As a consequence, common pathways would not be highlighted as association signals. It is important to note that in a recent study, an association was found with RA in the extended genomic segment including *TRAF1 *but excluding the *C5 *coding region [31]. Therefore, more specific and potentially unlinked SNP markers may exist and should be taken into account.

We only found nominal significance for SNPs rs10499194_*OLIG3/TNFAIP3 *_and rs6920220_*OLIG3/TNFAIP3*_, identified by Plenge and colleagues as independent RA risk markers [6]. The two SNPs are located on chromosome 6q23 and are in weak LD. SNP rs10499194_*OLIG3/TNFAIP3 *_showed a pronounced effect on RA risk in a recessive model in our study sample (*P *= 0.014), and, hence, might need larger populations to be detected with study-wide significance. Interestingly, minor allele frequency for rs10499194_*OLIG3/TNFAIP3 *_(0.315) is on the upper end whereas that for rs6920220_*OLIG3/TNFAIP3 *_(0.154) is below the frequencies from previously published studies [6,7]. Again, this may be caused by our study design or represent an ethnical characteristic. Perfect proxies of rs10499194_*OLIG3/TNFAIP3 *_are also associated with a risk of systemic lupus erythematosus [32]. Therefore, this genomic region might contribute to risk for autoimmune diseases and needs to be analyzed in further studies with higher power to detect an effect.

We were not able to show an association between the six SNPs and RA in the male subset of our population, which was likely to be due to a lack of power. However, gender-specific influence on association signal can not be excluded. Recently, in the Wellcome Trust Case Control Consortium genome-wide association study, a single SNP (rs11761231) generated a strong signal in the gender-differentiated analyses for RA, with an additive effect in females and no effect in males [4]. In contrast, a protective effect of the HLA-DRB1*13 allele was obvious in our male subgroup with an OR of 0.32 (i.e. OR = 3.13 for susceptibility allele). One possible explanation is the moderate SNP OR between 1.38 and 1.67 in the whole sample and, therefore, a loss of power to detect this effect in the small male sample (87 cases, 60 controls).

Several limitations of our study have to be considered. The summarization of all HLA-DRB1*01xx and *04xx alleles as SE alleles ignored the protective effects of *0103 and *0402 and the neutral effect of *0403, *0406, and *0407 subtypes. However, a recent report by Morgan and colleagues showed that the frequency of these alleles is very low [20]. Therefore, we may have underestimated the risk effect of HLA-DRB1*01 and *04 alleles in this study but confirmed the association between HLA-DRB1*04 SE and RA.

Our RA population is heterogenous in relation to RF and anti-CCP. Another study showed that the *HLA-DRB1 *SE alleles are only associated with anti-CCP-positive RA in a European population, where the combination of smoking history and SE alleles increased the risk for RA 21-fold [33]. Here, we found significant association to RA risk for *PTPN22 *variant rs2476601 and *HLA-DRB1 *alleles in anti-CCP-positive RA patients compared with OA controls. Analysis within our RA group divided into anti-CCP-positive and anti-CCP-negative subgroups revealed a pattern of association for *HLA-DRB1*-alleles similar to that found in the unstratified case-control setting. It remains unclear whether we had too little power to detect other effects or in fact found a significant causal interaction between serum anti-CCP antibody, *HLA-DRB1 *alleles, and rs2476601_*PTPN22 *_as previously described [33,34].

The ascertainment strategy used here was not aimed at collecting special subgroups (e.g. only RF-positive RA cases with detectable anti-CCP) and, therefore, is not presenting a particular form of pathology with a higher power to detect specific genetic factors. However, this population reflects the clinical reality and, hence, allows a better risk assessment for the general patient with RA.

The predictive value of genetic markers for RA diagnosis is not obvious when using a limited number of alleles [35]. However, the knowledge of nearly all genetic variants contributing to both RA and OA susceptibility in a given ethnicity may help to prevent clinical mismanagement and avoid excessive costs. Our population is the first aimed at identifying genetic differences between RA and OA and, therefore, allowing the dissection of genetic markers for diagnosis in the border area between these two disease entities.

## Conclusions

Our study demonstrates strong evidence that polymorphisms in *HLA-DRB1*, *PTPN22*, and *STAT4 *genes contribute to RA susceptibility in a comprehensively characterized Slovak case population compared with a gender-matched OA control group.

## Abbreviations

CCP: cyclic citrullinated peptide; CI: confidence interval; ELISA: enzyme-linked immunosorbent assay; HLA: human leukocyte antigen; LD: linkage disequilibrium; OA: osteoarthritis; OR: odds ratio; PCR: polymerase chain reaction; RA: rheumatoid arthritis; RF: rheumatoid factor; SE: shared epitope; SNP: single nucleotide polymorphism.

## Competing interests

The authors declare that they have no competing interests.

## Authors' contributions

KS carried out the SNP genotyping and statistical analysis and drafted the manuscript. JR and SB collected the sample and phenotyped the patients. HGW and SF carried out the HLA typing. CH participated in study coordination and helped to draft the manuscript. RS conceived of the study, and participated in its design and coordination and helped to draft the manuscript. All authors read and approved the final manuscript.

## Supplementary Material

Additional file 1 Word file containing 18 tables. Table S1 lists the *HLA-DRB1 *allele classification. Table S2 lists the single nucleotide polymorphism (SNP) association results from different genetic models in rheumatoid arthritis (RA)- osteoarthritis (OA) case-control sample. Table S3 lists the SNP association analysis results in male RA case-control sample. Table S4 lists the SNP association analysis results in female RA-OA case-control sample. Table S5 lists the SNP association analysis results in RA patients with rheumatoid factor (RF) vs. RA patients without RF. Table S6 lists the SNP association analysis results in RA patients with RF vs. OA controls. Table S7 lists the SNP association analysis results in RA patients without RF vs. OA controls. Table S8 lists the SNP association analysis results in anti-cyclic citrullinated peptide (CCP)-positive RA patients vs. anti-CCP-negative RA patients. Table S9 lists the SNP association analysis results in anti-CCP-positive RA patients vs. anti-CCP-negative OA patients. Table S10 lists the SNP association analysis results in anti-CCP-negative RA patients vs. anti-CCP-negative OA patients. Table S11 lists the *HLA-DRB1 *association analysis results in male RA case-control sample. Table S12 lists the *HLA-DRB1 *association analysis results in female RA case-control sample. Table S13 lists the *HLA-DRB1 *association analysis results in RA patients with RF vs. RA patients without RF. Table S14 lists the *HLA-DRB1 *association analysis results in RA patients with RF vs. OA controls. Table S15 lists the *HLA-DRB1 *association analysis results in RA patients without RF vs. OA controls. Table S16 lists the *HLA-DRB1 *association analysis results in anti-CCP-positive RA patients vs. anti-CCP-negative RA patients. Table S17 lists the *HLA-DRB1 *association analysis results in anti-CCP-positive RA patients vs. anti-CCP-negative OA patients. Table S18 lists the *HLA-DRB1 *association analysis results in anti-CCP-negative RA patients vs. anti-CCP-negative OA patients. Click here for file
