# Supplementary material for: Association of common polymorphisms in known susceptibility genes with rheumatoid arthritis in a Slovak population using osteoarthritis patients as controls
Source: Arthritis Res Ther. 2009 May 15;11(3):R70. doi: 10.1186/ar2699 (PMC2714116; doi:10.1186/ar2699)
Supplement: Additional file 1 — Word file containing 18 tables. Table S1 lists the HLA-DRB1 allele classification. Table S2 lists the single nucleotide polymorphism (SNP) association results from different genetic models in rheumatoid arthritis (RA)- osteoarthritis (OA) case-control sample. Table S3 lists the SNP association analysis results in male RA case-control sample. Table S4 lists the SNP association analysis results in female RA-OA case-control sample. Table S5 lists the SNP association analysis results in RA patients with rheumatoid factor (RF) vs. RA patients without RF. Table S6 lists the SNP association analysis results in RA patients with RF vs. OA controls. Table S7 lists the SNP association analysis results in RA patients without RF vs. OA controls. Table S8 lists the SNP association analysis results in anti-cyclic citrullinated peptide (CCP)-positive RA patients vs. anti-CCP-negative RA patients. Table S9 lists the SNP association analysis results in anti-CCP-positive RA patients vs. anti-CCP-negative OA patients. Table S10 lists the SNP association analysis results in anti-CCP-negative RA patients vs. anti-CCP-negative OA patients. Table S11 lists the HLA-DRB1 association analysis results in male RA case-control sample. Table S12 lists the HLA-DRB1 association analysis results in female RA case-control sample. Table S13 lists the HLA-DRB1 association analysis results in RA patients with RF vs. RA patients without RF. Table S14 lists the HLA-DRB1 association analysis results in RA patients with RF vs. OA controls. Table S15 lists the HLA-DRB1 association analysis results in RA patients without RF vs. OA controls. Table S16 lists the HLA-DRB1 association analysis results in anti-CCP-positive RA patients vs. anti-CCP-negative RA patients. Table S17 lists the HLA-DRB1 association analysis results in anti-CCP-positive RA patients vs. anti-CCP-negative OA patients. Table S18 lists the HLA-DRB1 association analysis results in anti-CCP-negative RA patients vs. anti-CCP-negative OA patient [file ar2699-S1.doc]

Table S1. *HLA-DRB1* allele classification.

| *HLA-DRB1* allele | Amino acid sequence at position 67 70 71 72 73 74 | Classification ^a^ |
| --- | --- | --- |
| *01 | [I/L] [D/Q] [E/R] R A A | SE ^b^ |
| *03 | L Q K R G R | N |
| *04 | [I/L] [D/Q] [E/K/R] R A [A/E] | SE ^b^ |
| *07 | I D R R G Q | P |
| *08 | [F/I] D R R A L | N ^c^ |
| *09 | F R R R A E | N |
| *10 | L R R R A A | SE |
| *11 | [F/I] D [E/R] R A A | N ^c^ |
| *12 | [F/I] D R R A A | P ^c^ |
| *13 | [F/I] D [E/K/R] R A A | P ^c^ |
| *14 | L [Q/R] R R A [A/E] | N ^c^ |
| *15 | [F/I] Q A R A A | P ^c^ |
| *16 | [F/L] D R R A A | N ^c^ |

^a^ according to de Vries et al. [19]

^b^ simplification as indicated in text

^c^ simplification according to Morgan et al. [20]

SE, shared epitope; N, neutral; P, protective

Table S2. SNP association results from different genetic models in RA-OA case-control sample.

|  |  | Genotypic | Dominant ^a^ | Recessive ^b^ | Additive |
| --- | --- | --- | --- | --- | --- |
| SNP |  | *P* | *P* | *P* | *P* |
| rs2476601 |  | n. a. | n. a. | n. a. | 8.8*10^-4^ |
| rs7574865 |  | 7.7*10^-5^ | 2.1*10^-5^ | 0.020 | 2.1*10^-5^ |
| rs10499194 |  | 0.041 | 0.175 | 0.014 | 0.034 |
| rs6920220 |  | 0.046 | 0.013 | 0.530 | 0.017 |
| rs3761847 |  | 0.674 | 0.380 | 0.862 | 0.488 |
| rs10818488 |  | 0.787 | 0.519 | 0.990 | 0.663 |

^a^ Genotypes 22 + 12 vs. 11

^b^ Genotypes 22 vs. 12 + 11

Numbers of genotypes (11, 12, 22) according to alleles from Table 2; n. a., not applicable due to low cell count (<5) in χ² test

Table S3. SNP association analysis results in male RA case-control sample.

|  | RA case genotypes | | | | |  | RA-free OA control genotypes | | | | |  | Allelic | Allelic OR |  |
| --- | --- | --- | --- | --- | --- | --- | --- | --- | --- | --- | --- | --- | --- | --- | --- |
| SNP | 11 | 12 | 22 | MAF | *P* (HWE) |  | 11 | 12 | 22 | MAF | *P* (HWE) |  | *P* | (95% CI) | Locus |
| rs2476601 | 63 | 21 | 2 | 0.145 | 1 |  | 47 | 13 | 0 | 0.108 | 1 |  | 0.355 | 1.40 (0.68-2.86) | *PTPN22* |
| rs7574865 | 46 | 37 | 2 | 0.241 | 0.135 |  | 35 | 24 | 1 | 0.217 | 0.264 |  | 0.626 | 1.15 (0.66-2.01) | *STAT4* |
| rs10499194 | 44 | 38 | 5 | 0.276 | 0.590 |  | 36 | 19 | 5 | 0.242 | 0.296 |  | 0.512 | 1.20 (0.70-2.04) | *OLIG3/TNFAIP3* |
| rs6920220 | 52 | 30 | 3 | 0.212 | 0.753 |  | 43 | 15 | 1 | 0.144 | 1 |  | 0.149 | 1.60 (0.85-3.00) | *OLIG3/TNFAIP3* |
| rs3761847 | 29 | 39 | 17 | 0.429 | 0.657 |  | 14 | 38 | 8 | 0.450 | 0.041 |  | 0.728 | 0.92 (0.57-1.47) | *TRAF1/C5* |
| rs10818488 | 30 | 39 | 17 | 0.424 | 0.512 |  | 14 | 37 | 8 | 0.449 | 0.065 |  | 0.676 | 0.90 (0.56-1.45) | *TRAF1/C5* |

MAF: minor allele frequency; HWE, Hardy-Weinberg equilibrium; numbers of genotypes (11, 12, 22) according to alleles from Table 2

Table S4. SNP association analysis results in female RA-OA case-control sample.

|  | RA case genotypes | | | | |  | RA-free OA control genotypes | | | | |  | Allelic | Allelic OR |  |
| --- | --- | --- | --- | --- | --- | --- | --- | --- | --- | --- | --- | --- | --- | --- | --- |
| SNP | 11 | 12 | 22 | MAF | *P* (HWE) |  | 11 | 12 | 22 | MAF | *P* (HWE) |  | *P* | (95% CI) | Locus |
| rs2476601 | 293 | 123 | 12 | 0.172 | 1 |  | 192 | 48 | 2 | 0.107 | 1 |  | 1.5*10^-3^ | 1.72 (1.23-2.42) | *PTPN22* |
| rs7574865 | 213 | 168 | 52 | 0.314 | 0.044 |  | 161 | 63 | 16 | 0.198 | 0.013 |  | 4.6*10^-6^ | 1.86 (1.42-2.42) | *STAT4* |
| rs10499194 | 237 | 162 | 32 | 0.262 | 0.536 |  | 113 | 97 | 32 | 0.333 | 0.147 |  | 6.1*10^-3^ | 0.71 (0.56-0.91) | *OLIG3/TNFAIP3* |
| rs6920220 | 272 | 145 | 13 | 0.199 | 0.289 |  | 170 | 63 | 6 | 0.157 | 1 |  | 0.058 | 1.33 (0.99-1.80) | *OLIG3/TNFAIP3* |
| rs3761847 | 157 | 204 | 70 | 0.399 | 0.841 |  | 103 | 95 | 41 | 0.370 | 0.026 |  | 0.301 | 1.13 (0.90-1.42) | *TRAF1/C5* |
| rs10818488 | 156 | 201 | 68 | 0.397 | 0.839 |  | 102 | 97 | 42 | 0.376 | 0.028 |  | 0.451 | 1.09 (0.87-1.38) | *TRAF1/C5* |

MAF: minor allele frequency; HWE, Hardy-Weinberg equilibrium; numbers of genotypes (11, 12, 22) according to alleles from Table 2

Table S5. SNP association analysis results in RA patients with RF (*n* = 280) vs. RA patients without RF (*n* = 228).

| SNP |  | Allelic *P* | Allelic OR (95% CI) | Locus |
| --- | --- | --- | --- | --- |
| rs2476601 |  | 0.947 | 0.99 (0.71-1.38) | *PTPN22* |
| rs7574865 |  | 0.796 | 1.04 (0.79-1.36) | *STAT4* |
| rs10499194 |  | 0.986 | 1.00 (0.76-1.33) | *OLIG3/TNFAIP3* |
| rs6920220 |  | 0.856 | 0.97 (0.71-1.33) | *OLIG3/TNFAIP3* |
| rs3761847 |  | 0.153 | 1.20 (0.93-1.55) | *TRAF1/C5* |
| rs10818488 |  | 0.154 | 1.20 (0.93-1.55) | *TRAF1/C5* |

Table S6. SNP association analysis results in RA patients with RF (*n* = 280) vs. OA controls (*n* = 303).

| SNP |  | Allelic *P* | Allelic OR (95% CI) | Locus |
| --- | --- | --- | --- | --- |
| rs2476601 |  | 5.3*10^-3^ | 1.62 (1.15-2.29) | *PTPN22* |
| rs7574865 |  | 4.6*10^-5^ | 1.74 (1.33-2.28) | *STAT4* |
| rs10499194 |  | 0.075 | 0.79 (0.62-1.02) | *OLIG3/TNFAIP3* |
| rs6920220 |  | 0.059 | 1.34 (0.99-1.82) | *OLIG3/TNFAIP3* |
| rs3761847 |  | 0.208 | 1.16 (0.92-1.47) | *TRAF1/C5* |
| rs10818488 |  | 0.300 | 1.13 (0.89-1.43) | *TRAF1/C5* |

Table S7. SNP association analysis results in RA patients without RF (*n* = 228) vs. OA controls (*n* = 303).

| SNP |  | Allelic *P* | Allelic OR (95% CI) | Locus |
| --- | --- | --- | --- | --- |
| rs2476601 |  | 6.2*10^-3^ | 1.64 (1.15-2.35) | *PTPN22* |
| rs7574865 |  | 2.9*10^-4^ | 1.68 (1.27-2.23) | *STAT4* |
| rs10499194 |  | 0.090 | 0.79 (0.60-1.04) | *OLIG3/TNFAIP3* |
| rs6920220 |  | 0.047 | 1.38 (1.00-1.90) | *OLIG3/TNFAIP3* |
| rs3761847 |  | 0.793 | 0.97 (0.75-1.24) | *TRAF1/C5* |
| rs10818488 |  | 0.634 | 0.94 (0.73-1.21) | *TRAF1/C5* |

Table S8. SNP association analysis results in anti-CCP-positive RA patients (*n* = 239) vs. anti-CCP-negative RA patients (*n* = 65).

| SNP |  | Allelic *P* | Allelic OR (95% CI) | Locus |
| --- | --- | --- | --- | --- |
| rs2476601 |  | 0.067 | 1.72 (0.96-3.09) | *PTPN22* |
| rs7574865 |  | 0.698 | 0.92 (0.60-1.40) | *STAT4* |
| rs10499194 |  | 0.766 | 1.07 (0.69-1.67) | *OLIG3/TNFAIP3* |
| rs6920220 |  | 0.042 | 1.75 (1.01-3.01) | *OLIG3/TNFAIP3* |
| rs3761847 |  | 0.617 | 0.90 (0.61-1.35) | *TRAF1/C5* |
| rs10818488 |  | 0.706 | 0.93 (0.62-1.38) | *TRAF1/C5* |

Table S9. SNP association analysis results in anti-CCP-positive RA patients (*n* = 239) vs. anti-CCP-negative OA patients (*n* = 120).

| SNP |  | Allelic *P* | Allelic OR (95% CI) | Locus |
| --- | --- | --- | --- | --- |
| rs2476601 |  | 4.2*10^-4^ | 2.42 (1.46-4.01) | *PTPN22* |
| rs7574865 |  | 0.100 | 1.35 (0.94-1.92) | *STAT4* |
| rs10499194 |  | 0.263 | 0.82 (0.58-1.16) | *OLIG3/TNFAIP3* |
| rs6920220 |  | 0.074 | 1.45 (0.96-2.17) | *OLIG3/TNFAIP3* |
| rs3761847 |  | 0.808 | 1.03 (0.74-1.41) | *TRAF1/C5* |
| rs10818488 |  | 0.975 | 0.99 (0.72-1.37) | *TRAF1/C5* |

Table S10. SNP association analysis results in anti-CCP-negative RA patients (*n* = 65) vs. anti-CCP-negative OA patients (*n* = 120).

| SNP |  | Allelic *P* | Allelic OR (95% CI) | Locus |
| --- | --- | --- | --- | --- |
| rs2476601 |  | 0.336 | 1.41 (0.70-2.84) | *PTPN22* |
| rs7574865 |  | 0.116 | 1.46 (0.91-2.35) | *STAT4* |
| rs10499194 |  | 0.284 | 0.77 (0.48-1.25) | *OLIG3/TNFAIP3* |
| rs6920220 |  | 0.541 | 0.83 (0.45-1.52) | *OLIG3/TNFAIP3* |
| rs3761847 |  | 0.573 | 1.14 (0.73-1.76) | *TRAF1/C5* |
| rs10818488 |  | 0.748 | 1.08 (0.69-1.67) | *TRAF1/C5* |

Table S11. *HLA-DRB1* association analysis results in male RA case-control sample.

|  | RA case genotypes ^b^ | | | | |  | RA-free OA control genotypes ^b^ | | | | |  | Allelic | Allelic OR |
| --- | --- | --- | --- | --- | --- | --- | --- | --- | --- | --- | --- | --- | --- | --- |
| *HLA-DRB1* allele ^a^ | 0 | 1 | 2 | MAF | *P* (HWE) |  | 0 | 1 | 2 | MAF | *P* (HWE) |  | *P* | (95% CI) |
| *01 | 56 | 27 | 1 | 0.173 | 0.446 |  | 48 | 11 | 1 | 0.108 | 0.517 |  | 0.128 | 1.72 (0.85-3.46) |
| *03 | 76 | 6 | 2 | 0.060 | 0.021 |  | 50 | 10 | 0 | 0.083 | 1 |  | 0.433 | 0.70 (0.28-1.73) |
| *04 | 49 | 29 | 6 | 0.244 | 0.558 |  | 47 | 12 | 1 | 0.117 | 0.576 |  | 6.7*10^-3^ | 2.44 (1.26-4.73) |
| *07 | 64 | 20 | 0 | 0.119 | 0.595 |  | 47 | 12 | 1 | 0.117 | 0.576 |  | 0.951 | 1.02 (0.49-2.12) |
| *08 | 82 | 2 | 0 | 0.012 | 1 |  | 54 | 6 | 0 | 0.050 | 1 |  | 0.052 | 0.23 (0.05-1.15) |
| *09 | 81 | 3 | 0 | 0.018 | 1 |  | 59 | 1 | 0 | 0.008 | 1 |  | 0.496 | 2.16 (0.22-21.1) |
| *10 | 84 | 0 | 0 | 0 | n. a. |  | 60 | 0 | 0 | 0 | n. a. |  | n. a. | n. a. |
| *11 | 64 | 18 | 2 | 0.131 | 0.623 |  | 51 | 8 | 1 | 0.083 | 0.335 |  | 0.205 | 1.66 (0.75-3.64) |
| *12 | 83 | 1 | 0 | 0.006 | 1 |  | 55 | 5 | 0 | 0.042 | 1 |  | 0.036 | 0.14 (0.02-1.19) |
| *13 | 75 | 8 | 1 | 0.060 | 0.247 |  | 41 | 18 | 1 | 0.167 | 1 |  | 3.3*10^-3^ | 0.32 (0.14-0.70) |
| *14 | 82 | 2 | 0 | 0.012 | 1 |  | 58 | 1 | 1 | 0.025 | 0.025 |  | 0.402 | 0.47 (0.08-2.86) |
| *15 | 67 | 16 | 1 | 0.107 | 1 |  | 47 | 12 | 1 | 0.117 | 0.576 |  | 0.800 | 0.91 (0.43-1.91) |
| *16 | 74 | 10 | 0 | 0.060 | 1 |  | 50 | 10 | 0 | 0.083 | 1 |  | 0.433 | 0.70 (0.28-1.72) |

^a^ Allele numbering according to Table S1 in Additional data file 1.

^b^ Numbers indicate counts of rare alleles.

MAF: minor allele frequency; HWE, Hardy-Weinberg equilibrium; n. a., not applicable

Table S12. *HLA-DRB1* association analysis results in female RA case-control sample.

|  | RA case genotypes ^b^ | | | | |  | RA-free OA control genotypes ^b^ | | | | |  | Allelic | Allelic OR |
| --- | --- | --- | --- | --- | --- | --- | --- | --- | --- | --- | --- | --- | --- | --- |
| *HLA-DRB1* allele ^a^ | 0 | 1 | 2 | MAF | *P* (HWE) |  | 0 | 1 | 2 | MAF | *P* (HWE) |  | *P* | (95% CI) |
| *01 | 294 | 112 | 6 | 0.151 | 0.249 |  | 175 | 64 | 0 | 0.134 | 0.011 |  | 0.412 | 1.15 (0.83-1.59) |
| *03 | 336 | 75 | 1 | 0.093 | 0.236 |  | 201 | 35 | 3 | 0.086 | 0.393 |  | 0.642 | 1.10 (0.74–1.63) |
| *04 | 219 | 163 | 30 | 0.271 | 1 |  | 187 | 52 | 0 | 0.109 | 0.087 |  | 5.3*10^-12^ | 3.04 (2.19-4.21) |
| *07 | 350 | 62 | 0 | 0.075 | 0.152 |  | 177 | 57 | 5 | 0.140 | 0.791 |  | 1.6*10^-4^ | 0.50 (0.35-0.72) |
| *08 | 389 | 23 | 0 | 0.028 | 1 |  | 225 | 14 | 0 | 0.029 | 1 |  | 0.886 | 0.95 (0.49-1.87) |
| *09 | 404 | 8 | 0 | 0.010 | 1 |  | 237 | 2 | 0 | 0.004 | 1 |  | 0.271 | 2.33 (0.49-11.0) |
| *10 | 382 | 30 | 0 | 0.036 | 1 |  | 234 | 5 | 0 | 0.010 | 1 |  | 5.3*10^-3^ | 3.57 (1.38-9.28) |
| *11 | 339 | 70 | 3 | 0.092 | 1 |  | 178 | 60 | 1 | 0.130 | 0.144 |  | 0.034 | 0.68 (0.48-0.97) |
| *12 | 403 | 9 | 0 | 0.011 | 1 |  | 227 | 12 | 0 | 0.025 | 1 |  | 0.050 | 0.43 (0.18-1.03) |
| *13 | 359 | 51 | 2 | 0.067 | 0.699 |  | 185 | 51 | 3 | 0.119 | 1 |  | 1.1*10^-3^ | 0.53 (0.36-0.78) |
| *14 | 393 | 19 | 0 | 0.023 | 1 |  | 225 | 14 | 0 | 0.029 | 1 |  | 0.491 | 0.78 (0.39-1.58) |
| *15 | 350 | 58 | 4 | 0.080 | 0.316 |  | 181 | 56 | 2 | 0.126 | 0.551 |  | 7.5*10^-3^ | 0.61 (0.42-0.88) |
| *16 | 361 | 50 | 1 | 0.063 | 1 |  | 212 | 26 | 1 | 0.059 | 0.568 |  | 0.743 | 1.08 (0.67-1.74) |

^a^ Allele numbering according to Table S1 in Additional data file 1.

^b^ Numbers indicate counts of rare alleles.

MAF: minor allele frequency; HWE, Hardy-Weinberg equilibrium

Table S13. *HLA-DRB1* association analysis results in RA patients with RF (*n* = 263) vs. RA patients without RF (*n* = 221).

| *HLA-DRB1* allele ^a^ |  | Allelic *P* | Allelic OR (95% CI) |
| --- | --- | --- | --- |
| *01 |  | 0.788 | 0.95 (0.67-1.35) |
| *03 |  | 0.680 | 1.10 (0.70-1.72) |
| *04 |  | 1.4*10^-3^ | 1.61 (1.20-2.16) |
| *07 |  | 1.4*10^-3^ | 0.46 (0.28-0.75) |
| *08 |  | 0.326 | 1.51 (0.66-3.45) |
| *09 |  | 0.782 | 0.84 (0.24-2.92) |
| *10 |  | 0.108 | 1.90 (0.86-4.22) |
| *11 |  | 0.037 | 0.64 (0.42-0.98) |
| *12 |  | 0.120 | 0.36 (0.09-1.39) |
| *13 |  | 0.059 | 0.62 (0.37-1.02) |
| *14 |  | 0.482 | 1.38 (0.56-3.35) |
| *15 |  | 0.571 | 1.14 (0.72-1.80) |
| *16 |  | 0.935 | 1.02 (0.61-1.71) |

^a^ Allele numbering according to Table S1 in Additional data file 1.

Table S14. *HLA-DRB1* association analysis results in RA patients with RF (*n* = 263) vs. OA controls (*n* = 299).

| *HLA-DRB1* allele ^a^ |  | Allelic *P* | Allelic OR (95% CI) |
| --- | --- | --- | --- |
| *01 |  | 0.260 | 1.21 (0.87-1.70) |
| *03 |  | 0.725 | 1.08 (0.71-1.63) |
| *04 |  | 3.5*10^-16^ | 3.56 (2.59-4.88) |
| *07 |  | 3.4*10^-6^ | 0.36 (0.23-0.56) |
| *08 |  | 0.774 | 0.91 (0.46-1.77) |
| *09 |  | 0.372 | 1.90 (0.45-8.00) |
| *10 |  | 7.7*10^-4^ | 4.69 (1.75-12.6) |
| *11 |  | 0.033 | 0.65 (0.44-0.97) |
| *12 |  | 4.0*10^-3^ | 0.20 (0.06-0.67) |
| *13 |  | 1.4*10^-5^ | 0.38 (0.24-0.60) |
| *14 |  | 0.700 | 0.87 (0.42-1.80) |
| *15 |  | 0.063 | 0.69 (0.47-1.02) |
| *16 |  | 0.940 | 1.02 (0.63-1.64) |

^a^ Allele numbering according to Table S1 in Additional data file 1.

Table S15. *HLA-DRB1* association analysis results in RA patients without RF (*n* = 221) vs. OA controls (*n* = 299).

| *HLA-DRB1* allele ^a^ |  | Allelic *P* | Allelic OR (95% CI) |
| --- | --- | --- | --- |
| *01 |  | 0.175 | 1.27 (0.90-1.81) |
| *03 |  | 0.928 | 0.98 (0.63-1.53) |
| *04 |  | 4.1*10^-6^ | 2.21 (1.57-3.11) |
| *07 |  | 0.194 | 0.78 (0.53-1.14) |
| *08 |  | 0.205 | 0.60 (0.27-1.33) |
| *09 |  | 0.251 | 2.27 (0.54-9.55) |
| *10 |  | 0.097 | 2.47 (0.82-7.41) |
| *11 |  | 0.931 | 1.02 (0.70-1.48) |
| *12 |  | 0.181 | 0.55 (0.23-1.34) |
| *13 |  | 0.022 | 0.62 (0.41-0.93) |
| *14 |  | 0.282 | 0.63 (0.27-1.47) |
| *15 |  | 0.020 | 0.61 (0.40-0.93) |
| *16 |  | 0.990 | 1.00 (0.60-1.65) |

^a^ Allele numbering according to Table S1 in Additional data file 1.

Table S16. *HLA-DRB1* association analysis results in anti-CCP-positive RA patients (*n* = 224) vs. anti-CCP-negative RA patients (*n* = 62).

| *HLA-DRB1* allele ^a^ |  | Allelic *P* | Allelic OR (95% CI) |
| --- | --- | --- | --- |
| *01 |  | 0.293 | 1.38 (0.76-2.50) |
| *03 |  | 0.096 | 0.58 (0.30-1.11) |
| *04 |  | 1.9*10^-3^ | 2.27 (1.34-3.86) |
| *07 |  | 0.766 | 1.12 (0.53-2.40) |
| *08 |  | 0.640 | 0.76 (0.24-2.43) |
| *09 |  | n. a. | n. a. |
| *10 |  | 0.456 | 1.60 (0.46-5.54) |
| *11 |  | 0.024 | 0.50 (0.27-0.92) |
| *12 |  | 7.3*10^-3^ | 0.14 (0.02-0.75) |
| *13 |  | 2.8*10^-3^ | 0.36 (0.18-0.72) |
| *14 |  | 0.031 | 0.31 (0.10-0.95) |
| *15 |  | 0.780 | 0.91 (0.47-1.75) |
| *16 |  | 0.037 | 3.32 (1.10-11.0) |

^a^ Allele numbering according to Table S1 in Additional data file 1; n. a., not applicable (no allele in either cases or controls).

Table S17. *HLA-DRB1* association analysis results in anti-CCP-positive RA patients (*n* = 224) vs. anti-CCP-negative OA patients (*n* = 120).

| *HLA-DRB1* allele ^a^ |  | Allelic *P* | Allelic OR (95% CI) |
| --- | --- | --- | --- |
| *01 |  | 0.228 | 1.33 (0.84-2.10) |
| *03 |  | 0.662 | 0.88 (0.49-1.57) |
| *04 |  | 2.1*10^-7^ | 3.12 (2.00-4.86) |
| *07 |  | 2.9*10^-3^ | 0.48 (0.30-0.79) |
| *08 |  | 0.078 | 0.48 (0.21-1.11) |
| *09 |  | 0.248 | 3.26 (0.39-27.2) |
| *10 |  | n. a. | n. a. |
| *11 |  | 0.099 | 0.64 (0.38-1.09) |
| *12 |  | 2.6*10^-3^ | 0.13 (0.03-0.62) |
| *13 |  | 7.6*10^-3^ | 0.44 (0.24-0.82) |
| *14 |  | 0.132 | 0.46 (0.17-1.29) |
| *15 |  | 0.247 | 0.75 (0.46-1.23) |
| *16 |  | 0.381 | 1.33 (0.70-2.54) |

^a^ Allele numbering according to Table S1 in Additional data file 1; n. a., not applicable (no allele in either cases or controls).

Table S18. *HLA-DRB1* association analysis results in anti-CCP-negative RA patients (*n* = 62) vs. anti-CCP-negative OA patients (*n* = 120).

| *HLA-DRB1* allele ^a^ |  | Allelic *P* | Allelic OR (95% CI) |
| --- | --- | --- | --- |
| *01 |  | 0.912 | 0.96 (0.50-1.87) |
| *03 |  | 0.248 | 1.51 (0.75-3.07) |
| *04 |  | 0.324 | 1.37 (0.73-2.57) |
| *07 |  | 0.026 | 0.43 (0.20-0.92) |
| *08 |  | 0.434 | 0.63 (0.20-2.01) |
| *09 |  | n. a. | n. a. |
| *10 |  | n. a. | n. a. |
| *11 |  | 0.438 | 1.29 (0.68-2.43) |
| *12 |  | 0.957 | 0.97 (0.29-3.28) |
| *13 |  | 0.540 | 1.24 (0.62-2.46) |
| *14 |  | 0.479 | 1.48 (0.50-4.35) |
| *15 |  | 0.572 | 0.82 (0.41-1.64) |
| *16 |  | 0.144 | 0.40 (0.11-1.42) |

^a^ Allele numbering according to Table S1 in Additional data file 1; n. a., not applicable (no allele in either cases or controls).
